# Therapy response prediction of focal cortex stimulation based on clinical parameters: a multicentre, non-interventional study protocol

**DOI:** 10.1136/bmjopen-2024-089903

**Published:** 2025-02-16

**Authors:** Sotirios Kalousios, Jürgen Hesser, Matthias Dümpelmann, Christoph Baumgartner, Hajo M Hamer, Martin Hirsch, Lukas Imbach, Elisabeth Kaufmann, Josua Kegele, Susanne Knake, Georg Leonhardt, Thomas Mayer, Ann Mertens, Ekaterina Pataraia, Felix von Podewils, Carlos M Quesada, Bernhard J Steinhoff, Rainer Surges, Berthold R Voges, Jan Wagner, Yvonne G Weber, Tim Wehner, Yaroslav Winter, Andreas Schulze-Bonhage

**Affiliations:** 1 Epilepsy Center, Neurocenter, University Medical Center, University of Freiburg, Freiburg, Germany; 2 Mannheim Institute for Intelligent Systems in Medicine, Department of Medicine Mannheim, Interdisciplinary Center for Scientific Computing (IWR), Interdisciplinary Center for Computer Engineering (ZITI), CZS Heidelberg Center for Model-Based AI, Heidelberg University, Heidelberg, Germany; 3 Department of Neurology, Clinic Hietzing, Vienna, Austria; 4 Karl Landsteiner Institute for Clinical Epilepsy Research & Cognitive Neurology, Vienna, Austria; 5 Epilepsy Center, Department of Neurology, University Hospital Erlangen, Friedrich-Alexander-University Erlangen-Nürnberg, Germany, Full member of ERN EpiCARE, Erlangen, Germany; 6 Swiss Epilepsy Center, Klinik Lengg, Zurich, Switzerland; 7 Epilepsy Center, Department of Neurology, LMU University Hospital, LMU Munich, Munich, Germany; 8 Department of Neurology and Epileptology, Hertie Institute of Clinical Brain Research, University of Tübingen, Hoppe-Seyler-Straße 3, 72076 Tübingen, Germany; 9 Department of Neurology, Epilepsy Center, University Hospital Marburg, Philipps-University Marburg, Marburg, Germany; 10 Technische Universität Dresden, Faculty of Medicine and University Hospital Carl Gustav Carus, Department of Neurosurgery, Fetscherstrasse 74, 01307 Dresden, Germany; 11 Epilepsy Center Kleinwachau, Radeberg, Germany; 12 Department of Neurology, 4Brain, Ghent University Hospital, Ghent, Belgium; 13 Department of Neurology, Medical University of Vienna, Vienna, Austria; 14 Comprehensive Center for Clinical Neurosciences & Mental Health, Medical University of Vienna, Vienna, Austria; 15 Department of Neurology, Epilepsy Center, University Medicine Greifswald, Greifswald, Germany; 16 Department of Neurology and Center for Translational and Behavioral Neurosciences (C-TNBS), University Medicine Essen, Essen, Germany; 17 Kork Epilepsy Center, Kehl-Kork, Germany; 18 Department of Epileptology, University Hospital Bonn, Bonn, Germany; 19 Department of Epileptology, Protestant Hospital Hamburg-Alsterdorf, Elisabeth-Flügge-Str.1, Hamburg, Germany; 20 Department of Neurology, University of Ulm and Universitäts- und Rehabilitationskliniken Ulm, Ulm, Germany; 21 Department of Epileptology and Neurology, RWTH Aachen University, Aachen, Germany; 22 Ruhr-Epileptology, Department of Neurology, University Hospital Knappschaftskrankenhaus Bochum, Ruhr-University Bochum, Bochum, Germany; 23 Department of Neurology, Mainz Comprehensive Epilepsy and Sleep Medicine Center, University Medical Center of the Johannes Gutenberg University, Mainz, Germany

**Keywords:** Patients, Epilepsy, NEUROLOGY, Electric Stimulation Therapy, Machine Learning

## Abstract

**ABSTRACT:**

**Introduction:**

A novel focal cortex stimulation (FCS) device has recently received approval in Europe for patients with focal drug-resistant epilepsy (DRE). After 6 months of stimulation, 17 of 32 patients achieved ≥50% reduction in seizure frequency compared with their prestimulation baseline (responders). Currently, there is no established method for predicting FCS treatment response prior to implantation.

**Methods and analysis:**

This is an ongoing combined retrospective-prospective non-interventional multicentre study. Clinical data of up to 100 patients treated with FCS are collected across 20 collaborating epilepsy centres in four European countries. The key outcome parameters, seizure frequency and severity, are measured along with metrics on cognition, mood and quality of life, both pre-electrode and postelectrode implantation. The data are complemented by demographics, medical history and information on antiseizure medication and FCS treatment parameters during the stimulation period. In addition to clinical data, MRI and electroencephalography registrations are used to gain insights into spatial and electrophysiological aspects of FCS. Multivariate statistical and machine learning analyses are employed to identify key predictive biomarkers associated with patient outcomes (responders vs non-responders). The primary goal is to improve counselling for DRE patients by identifying promising candidates for FCS treatment.

**Ethics and dissemination:**

This study has received approval from the ethics committee of the University of Freiburg, Germany (23–1540 S1; 23–1183_1-S1-retro). The same approval is applicable for all participating centres in Germany as part of a multicentre study. Ghent University Hospital, Belgium, has received approval for participation in the retrospective arm from their local ethics committee (ONZ-2024-0168). The final approvals for the participating Swiss and Austrian sites are still pending. The results will be made available to the public through peer-reviewed journals and conference presentations.

**STRENGTHS AND LIMITATIONS OF THIS STUDY**

- Multicentre, non-interventional study design assessing clinical practice and outcomes of focal cortex stimulation.
- Implementation of interpretable, state-of-the-art machine learning techniques for therapy response prediction.
- Inclusion of clinical data, stimulation parameters and raw data from MRI and electroencephalography recordings.
- Comprehensive assessment of outcomes, including seizure frequency, seizure severity and quality of life.
- Limited validity of self-reported seizure diaries.

## Introduction

 Epilepsy affects up to 1% of the general population,^1^ and despite advances in medical therapy, a third of people with epilepsy (PWE) do not achieve satisfactory seizure control.^2 3^ As a result, patients with pharmacoresistant epilepsy are often exposed to the physical, neurobiological, psychological and social implications tied to their condition.^4 5^ Patients with focal onset epilepsy may benefit from resective or ablative surgery,^6^ provided there is a single, clearly identifiable epileptogenic focus. For non-surgical candidates, neuromodulation is a further therapeutic option.^7^

A minimally invasive,^8^ implantable transcranial neurostimulation device was recently approved for patients with focal epilepsy in Europe and is now commercially available.^9^ The Epicranial Application of Stimulation Electrodes for Epilepsy (EASEE) system consists of a 5-channel array of electrodes, placed subgaleally over the individual epileptogenic brain focus. This novel neuromodulation concept is referred to as focal cortex stimulation (FCS). The device currently supports three stimulation modes. While direct current-like stimulation is applied periodically, high-frequency stimulation can be applied both periodically and on the patient’s demand.

Two prospective pilot clinical trials (A Pilot Study to Assess the Feasibility of Neurostimulation With the EASEE System to Treat Medically Refractory Focal Epilepsy [EASEE II] and A Pilot Study to Assess the Feasibility of Patient-Controlled Neurostimulation With the EASEE System to Treat Medically Refractory Focal Epilepsy [PIMIDES I]^10^) have shown the effectiveness of FCS in terms of reducing seizure burden. After the initial 6 months of the stimulation period, the pooled analysis showed that 17 of 32 participants (53.1%) responded to treatment with ≥50% reduction in seizure frequency compared with baseline (median reduction: 52%).^9^ No serious adverse events (SAEs) were considered to be associated with the surgical procedure, the device or stimulation at 8 months.^9^ Similarly, no surgical SAEs were reported for five patients implanted outside these trials.^8^ Mild adverse events considered related to implantation included transient pain or tingling at the skin incision sites. Other reported adverse events included five patients who experienced an increase in seizure frequency, one case of status epilepticus in a patient with a history before implantation, the onset of depressive symptoms in a patient with a pre-existing condition and one case attributed to COVID-19 restrictions.^9^

The efficacy and safety profile are comparable to other long-term neuromodulatory approaches, such as vagus nerve stimulation (VNS), deep brain stimulation of the anterior nucleus of the thalamus (ANT-DBS) and responsive neurostimulation (RNS), while being less invasive than the latter two. At 1 year follow-up, the VNS responder rate ranges from 31% to 49%, with a median or mean seizure reduction of 26%–52%.^7^ For ANT-DBS, the Stimulation of the Anterior Nucleus of the Thalamus for Epilepsy (SANTE) study reported a responder rate of 43%, with a median seizure reduction of 41% at 12 months, while the European Medtronic Registry for Epilepsy (MORE) registry reported a responder rate of 32.3% and a median seizure reduction of 33.1% at 24 months.^11 12^ For RNS, the responder rate after 12 months was 43%, with a median seizure reduction of 44%.^13 14^ Relevant adverse events include hoarseness (29%) and paresthesia (12%) for VNS and depression (15%), memory impairment (13%) and intracranial haemorrhage (5%) for ANT-DBS. For RNS, intracranial haemorrhage was reported in 12% of patients and headaches in 11%.^15^

These data suggest that FCS may offer an effective and safe adjunctive treatment option for adult patients with pharmacoresistant epilepsy with a predominant epileptic focus. However, the clinical parameters that determine how patients respond to the FCS treatment and the characteristics that differentiate responders from non-responders remain unclear.

The aim of the study is to predict the therapeutic effectiveness of FCS based on clinical parameters prior to implantation. Insights gained from this study could be crucial in identifying patients or patient groups that may benefit from this form of treatment. This, in turn, could enhance counselling and add to individually tailored therapeutic approaches in the future.

## Methods and analysis

### Study description and design

This is an ongoing combined retrospective-prospective non-interventional multicentre study initiated in January 2024. Clinical data of up to 100 PWE treated with FCS are being collected across 20 sites in four European countries (Austria, Belgium, Germany, Switzerland), with Freiburg Epilepsy Center as the coordinating centre.

Seven sites contribute previously acquired data from the EASEE II and PIMIDES I trials, forming the retrospective arm of the study (table 1). The prospective non-interventional arm includes 19 sites that are actively enrolling patients (table 2). The total duration of the study is 4 years.

**Table 1:** Participating sites in the retrospective study arm.

Retrospective arm	
Epilepsy Center, Neurocenter, University Medical Center, University of Freiburg, Freiburg, Germany	Department of Epileptology, University Hospital Bonn, Bonn, Germany
Mainz Comprehensive Epilepsy and Sleep Medicine Center, Department of Neurology, University Medical Center of the Johannes Gutenberg University, Mainz, Germany	Epilepsy Center, Department of Neurology, LMU University Hospital, LMU Munich, Munich, Germany
Department of Neurology and Epileptology, University Hospital Tübingen, Faculty of Medicine, University of Tübingen, Tübingen, Germany	Department of Neurology, Ghent University Hospital, Ghent, Belgium
Epilepsy Center, Department of Neurology, University Hospital Marburg, Philipps-University Marburg, Marburg, Germany	

**Table 2:** Participating sites in the prospective study arm.

Prospective arm	
Epilepsy Center, Neurocenter, University Medical Center, University of Freiburg, Freiburg, Germany	Swiss Epilepsy Center, Klinik Lengg, Zurich, Switzerland
Mainz Comprehensive Epilepsy and Sleep Medicine Center, Department of Neurology, University Medical Center of the Johannes Gutenberg University, Mainz, Germany	Department of Neurology, University of Ulm and Universitäts- und Rehabilitationskliniken Ulm, Ulm, Germany
Epilepsy Center, Department of Neurology, Friedrich-Alexander-University Erlangen-Nürnberg, Germany	Epilepsy Center, Department of Neurology, LMU University Hospital, LMU Munich, Munich, Germany
Epilepsy Center, Department of Neurology, University Medicine Greifswald, Greifswald, Germany	Department of Neurology, Clinic Hietzing, Vienna, Austria
Department of Neurology and Epileptology, University Hospital Tübingen, Faculty of Medicine, University of Tübingen, Tübingen, Germany	Ruhr-Epileptology, Department of Neurology, University Hospital Knappschaftskrankenhaus Bochum, Ruhr-University Bochum, Germany
Epilepsy Center, Department of Neurology, University Hospital Marburg, Philipps-University Marburg, Marburg, Germany	Department of Epileptology, Protestant Hospital Hamburg-Alsterdorf, Hamburg, Germany
Department of Neurosurgery, University Hospital Carl Gustav Carus, Technische Universität Dresden, Dresden, Germany	Kork Epilepsy Center, Kehl-Kork, Germany
Department of Epileptology, University Hospital Bonn, Bonn, Germany	Epilepsy Center Kleinwachau, Radeberg, Dresden, Germany
Department of Neurology, Medical University of Vienna, Vienna, Austria and Comprehensive Center for Clinical Neurosciences and Mental Health, Medical University of Vienna, Vienna, Austria	Epilepsy Center, Department of Neurology, University Medicine Essen, Essen, Germany
Department of Epileptology and Neurology, RWTH Aachen University, Aachen, Germany	

### Study population

The retrospective case series comprises 32 subjects who participated in the EASEE II and PIMIDES I pilot clinical trials^9^ that have been completed.

The prospective arm is non-interventional, aiming at the inclusion of approximately 70 patients treated with FCS. Study candidates are implanted with the EASEE device solely based on clinical decisions, independent of the study’s conduct.

### Inclusion criteria—prospective arm

1. PWE with a clinical diagnosis of focal epilepsy.
2. Patients aged above 18 years.
3. Pharmacoresistance to at least two antiseizure medications (ASMs).
4. FCS treatment with an EASEE system.
5. Written informed consent by the patient or legal guardian.

Similar criteria were applied in both clinical trials.^9 10^ All collaborating sites are responsible for patient selection and adherence to all eligibility criteria.

### Primary outcome

- To identify clinical parameters associated with good and moderate treatment responses, defined as a seizure reduction of ≥75% and ≥50%, respectively.

### Secondary outcomes

- Treatment efficacy—seizure frequency and severity.
- Effects on cognition, mood and quality of life (QoL).
- Quantification of stimulation-induced electroencephalography (EEG) changes—epileptiform activity and connectivity.

### Data collection

The retrospective arm uses data previously collected during the EASEE II and PIMIDES I clinical trials. In the prospective non-interventional phase, we exclusively collect data derived from routine clinical care at each site, both before and after electrode implantation. Consequently, there are no prespecified follow-up visits or stimulation protocols, as treatment is solely guided by clinical need. This approach ensures there is no additional risk or burden for the patients involved in the conduct of this study. The patients are followed throughout their individual stimulation treatment period, limited to a maximum of 3 years from the system activation date.

The collected data prior to implantation encompass demographic information, medical history, epilepsy aetiology, classification and duration. To later assess treatment efficacy and account for potential confounding factors, seizure frequency, seizure severity (SSQ)^16^ and current ASMs are collected during a ‘baseline period’ of up to 3 months. Seizure frequency is analysed through monthly seizure counts recorded in seizure diaries. This information is complemented by details about unsuccessful ASM trials or interventional treatment efforts. Additionally, we evaluate cognition, mood and QoL using the EpiTrack,^17^ Neurological Disorders Depression Inventory for Epilepsy (NDDI-E)^18 19^ and QoL in Epilepsy-Problems (QOLIE-31-P)^20^ questionnaires, respectively.

After implantation, we prospectively monitor disease burden, measured in seizure frequency, severity, accompanied by stimulation parameters and changes in ASM treatment. Treatment safety and adverse effects are monitored throughout the stimulation period, as well as effects on mood, cognition and QoL.

To better understand the spatial and neurophysiological aspects of FCS and their effect on outcome, MRI and EEG registrations are used, acquired both before and after stimulation.

The data of interest were specified by the clinical routine followed at the coordinating centre, and given the variation of clinical practice across sites and the non-interventional study design, data may be incomplete.

### Sample size calculation

This is an exploratory study for FCS treatment response, and the sample size calculation was drawn from previous literature that examined predictive biomarkers for other neurostimulation devices. A recent article on ANT-DBS identified significant differences in the theta oscillations between responders and non-responders in 15 patients.^21^ Similarly, global functional connectivity was found to be a response predictor in a cohort of 31 patients treated with an RNS system.^22^ Furthermore, a review of VNS^23^ included 28 articles reporting results from both retrospective and prospective studies.^24^ Sixteen putative predictive biomarkers were identified, with a median cohort size of 31 (range: 10–436). Based on this prior published work, we regard the inclusion of 100 participants as sufficient to achieve our primary and secondary outcomes.

### Data management

At each participating centre, all personal identifiers in the data are replaced by a unique patient ID (pseudonymisation). Additional precautions are implemented for MRI data, involving the removal of any facial features. Recruitment lists and de-identification logs are not shared between sites.

Clinical data can be entered directly into electronic case report forms (CRFs) managed within the REDCap^25^ database or submitted as physical CRFs to the Medical Center of Freiburg via postal service. MRI and EEG are securely transferred (encrypted) and stored on servers. Both the REDCap database and servers are hosted and administered by the Medical Center of Freiburg. Pseudonymised data are shared with the Mannheim Institute for Intelligent Systems in Medicine, University of Heidelberg, for data analysis.

### Data analysis

The Freiburg Epilepsy Center and the Mannheim Institute for Intelligent Systems in Medicine are jointly conducting data analyses. Patient classification (responders vs non-responders) will be correlated with clinical data in a pooled analysis. Both categorical and continuous variables, as well as EEG, will be examined with multivariate and univariate statistical analyses and machine learning techniques. Demographic information, epilepsy characteristics and secondary endpoints will be evaluated using descriptive statistics.

A newly developed MRI sequence is employed for electrode visualisation. Three-dimensional MRI data serve to model the applied stimulation fields based on the patients’ individual stimulation data and the brain and skull morphology. These models aim to illuminate the spatial parameters of FCS and their impact on outcomes. The focus lies on the EASEE electrode’s relative position to the lesion in MRI-positive cases, as well as the magnitude and angle of the electrical field within the region of interest. For MRI-negative cases, we will examine the relationship to the distribution of interictal and ictal epileptic EEG activity. Associations between lesion diameter and distance to the electrode will also be investigated.

EEG signals are used to quantify stimulation-induced changes in EEG epileptiform activity and alterations in connectivity. Specifically, changes in frequency and topography of epileptiform EEG discharges are assessed based on 10–20 and high-density EEG registrations.^26^ Local and global statistical network descriptors based on the mean phase coherence are used to analyse modulation in functional networks.^27^ Similar network analyses have already demonstrated their potential as prognostic factors for predicting seizure freedom in patients undergoing epilepsy surgery.^28^

**Figure 1:**
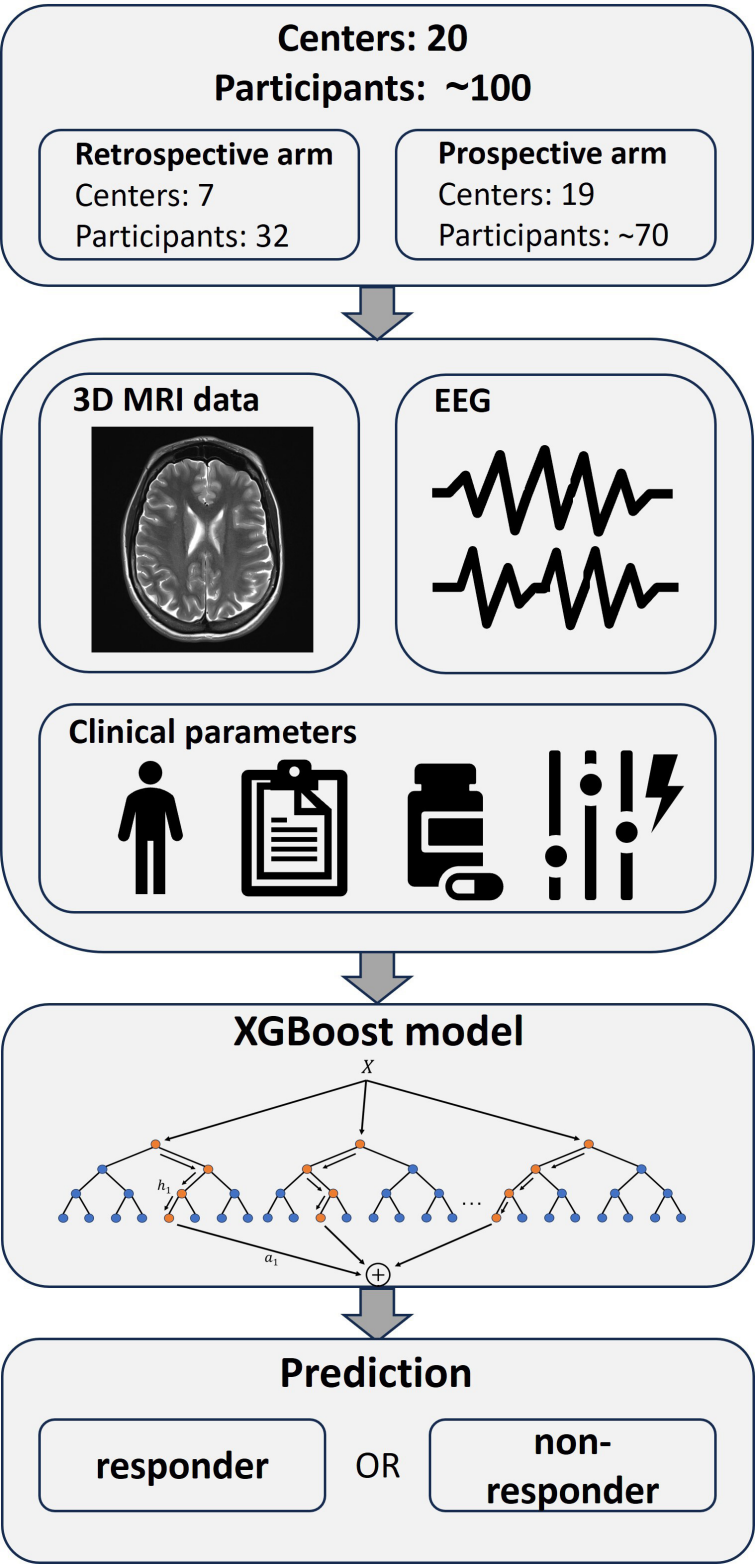
Flowchart of the therapy response prediction models. XGBoost, Extreme Gradient Boosting.

Finally, the Extreme Gradient Boosting^29^ algorithm will be used to construct predictive models for therapy response, using all available data, as well as to identify key predictive patient characteristics (figure 1).

### Patient and public involvement

The study aims to improve personalised counselling of patients, aligning with requests from international patient associations. The patient-based Christian-Homann Foundation supports the conduct of the study. Results will be shared with patient associations, and we will dedicate efforts to raise awareness about this new therapeutic strategy.

### Data availability statement

Patient consent does not include the transfer of personalised data outside the study consortium.

### Ethics and dissemination

The presented study has received approval from the ethics committee of the University of Freiburg, Germany (23-1540-S1; 23-1183_1-S1-retro) and will adhere to the principles of the Declaration of Helsinki and good clinical practice regulations. The same approval is applicable for all participating centres in Germany as part of a multicentre study. Ghent University Hospital, Belgium, has received approval for participation in the retrospective arm from their local ethics committee (ONZ-2024-0168). The final approvals for the participating Swiss and Austrian sites are still pending.

The outcomes of this study will be disseminated through publication in reputable international peer-reviewed journals as original research articles, where possible in an open-access format. Additionally, efforts will be concentrated on presenting the results at relevant national and international conferences and scientific meetings at various stages throughout the study timeline.
