# Supplementary material for: Therapy response prediction of focal cortex stimulation based on clinical parameters: a multicentre, non-interventional study protocol
Source: BMJ Open. 2025 Feb 16;15(2):e089903. doi: 10.1136/bmjopen-2024-089903 (PMC11873335; doi:10.1136/bmjopen-2024-089903)
Supplement: Supplementary file 1 [file bmjopen-2024-089903.reviewer_comments.pdf]

PEER REVIEW HISTORY

BMJ Open publishes all reviews undertaken for accepted manuscripts. Reviewers are asked to complete a checklist review form and are provided with free text boxes to elaborate on their assessment. These free text comments are reproduced below.

ARTICLE DETAILS

Title (Provisional)

Therapy response prediction of focal cortex stimulation based on clinical parameters – a multicenter, non-interventional study protocol

Authors

Kalousios, Sotirios; Hesser, Jürgen; Dümpelmann, Matthias; Baumgartner, Christoph; Hamer , Hajo; Hirsch, Martin; Imbach, Lukas; Kaufmann, Elisabeth; Kegele, Josua; Knake, Susanne; Leonhardt, Georg; Mayer, Thomas; Mertens, Ann; Pataraia, Ekaterina; von Podewils, Felix; Quesada, Carlos M.; Steinhoff, Bernhard J.; Surges, Rainer; Voges, Berthold R.; Wagner, Jan; Weber, Yvonne G.; Wehner, Tim; Winter, Yaroslav; Schulze-Bonhage, Andreas

---

VERSION 1 - REVIEW

**Reviewer**	**1**
**Name**	**Oane, I**
**Affiliation**	**University of Medicine and Pharmacy Carol Davila Bucharest**
**Date**	**22-Jul-2024**
**COI**	**none**

This is a very interesting study investigating the effect of direct cortical stimulation using different open-loop as well as on-demand stimulation protocols on seizure frequency in patients with drug-resistant epilepsy. Additionally, the authors aim to determine clinical and EEG biomarkers for responders.

I have a few comments:

Authors mention "electrode's relative position to the lesion" however they do not mention that all patients will be MRI positive. In cases of MRI negative where will the electrodes be placed?

At this point we know that epilepsy is a disease of connectivity and the epileptogenic lesion plays only a part in the epileptogenic network. Why did the authors focus only on the relation of FCS and the epileptogenic lesion?

Could the authors mention how they expect this type of stimulation to work in deep seated epileptogenic networks and how the study will deal with this limitation.

In the methods section it is unclear how the EEG data will be analyzed and integrated to serve as a biomarker. Could the authors describe in a more detailed manner the “data analysis” section?

Could the authors also provide additional information concerning the stimulation protocol, specifically when are the patients instructed to use the additional HFS stimulation.

**Reviewer**	**2**
**Name**	**Suller Marti, Ana**
**Affiliation**	**Western University, Clinical Neurological Sciences**
**Date**	**23-Jul-2024**
**COI**	**N/A**

It has been my pleasure to review this manuscript entitled Therapy response prediction of focal cortex stimulation based on clinical parameters – a multicenter, observational

study protocol’. This manuscript summarizes the protocol of an ongoing study using a new type of neuromodulation device called focal cortex stimulation. This device is used in patients with focal drug resistant epilepsy and requires the device to be implanted subgaleal over the epileptogenic focus. Overall this paper is nicely written manuscript. However, additional important information should be included.

In the abstract, if severity of seizures are evaluated, that information should be mentioned.

Regarding the keywords, neuromodulation should be included, as well as focal epilepsy.

Additional information regarding the procedure, complications and side effects should be included in the introduction. A clear explanation why FCS is better than open surgery or other neuromodulation devices should be mentioned, to make the case for new technologies. Furthermore, a better justification of why the proposed stimulation paradigms are selected needs to be incorporated into the manuscript.

In the study population section, a clear list of exclusion criteria should be included. Similarly, the duration of the follow ups for evaluating outcomes needs to be mentioned. It is mentioned that patients can modify ASM during the follow up period, please justify how you will control for ASM in the evaluation of the different outcomes.

In line 15 of study population, you mentioned this study is ‘non-interventional’. A clear explanation why this study was categorized as ‘non-interventional’ needs to be included in the manuscript.

An interesting part of the paper is the analysis of EEG and high-density EEG. Is High-density EEG a requirement? That may influence the results.

Please include how many patients will underwent to SEEG prior to FCS and how that may influence the FCS outcomes.

VERSION 1 - AUTHOR RESPONSE

Reviewer 1

This is a very interesting study investigating the effect of direct cortical stimulation using different open-loop as well as on-demand stimulation protocols on seizure frequency in patients with drug-resistant epilepsy. Additionally, the authors aim to determine clinical and EEG biomarkers for responders.

Comment/Question:
Authors mention "electrode's relative position to the lesion" however they do not mention that all patients will be MRI positive. In cases of MRI negative where will the electrodes be placed?

Response:
This is correct—both lesional and non-lesional patients are candidates for FCS, as reported in [9]. For the analyses conducted in this study, the electrode's position relative to the lesion can be determined in patients with lesions. For non-lesional patients, this is not feasible; however, a relationship to the distribution of interictal and ictal epileptic EEG activity can still be analyzed by including raw EEG data. This has now been additionally mentioned in the revision, stating: "For MRI-negative cases, we will examine the relationship to the distribution of interictal and ictal epileptic EEG activity" (Page 9, Data analysis).

Comment/Question:
At this point we know that epilepsy is a disease of connectivity and the epileptogenic lesion plays only a part in the epileptogenic network. Why did the authors focus only on the relation of FCS and the epileptogenic lesion?

Response:
We agree with the reviewer that also in focal epilepsies, connectivity of the epileptogenic lesion plays a crucial role. The neurostimulation approach chosen, however, focuses on modulation of the epileptogenic region, similar to responsive neurostimulation (RNS). A potential role of the epileptogenic network will be assessed when analyzing EEG recordings prior to and during stimulation, which may reflect network changes related to the focus stimulation. This includes the assessment of connectivity as a potential predictor of therapeutic response, and we will also explore the mid- and long-term effects of FCS on connectivity as part of our secondary study outcomes. To this aim, we will use local and global statistical network descriptors based on the mean phase coherence are utilized to analyze modulation in functional networks.

This is now clarified stating "Local and global statistical network descriptors based on the mean phase coherence are utilized to analyze modulation in functional networks[21]. Similar network analyses have already demonstrated their potential as prognostic factors for predicting seizure freedom in

patients undergoing epilepsy surgery" and by citing a recent work of our group on such connectivity features [22] in page 9 (Data analysis).

This comprehensive approach aims to deepen our understanding of how both lesion characteristics and network dynamics may influence treatment efficacy.

Comment/Question:
Could the authors mention how they expect this type of stimulation to work in deep seated epileptogenic networks and how the study will deal with this limitation.

Response:
In the clinical trials EASEE II and PIMIDES I, participants were required to have a neocortical focal epilepsy to be included. Deep-seated lesions, such as those in mesiotemporal epilepsy, were an exclusion criterion, and the effect of FCS has not been investigated.

In contrast, clinical use may also include more deep-seated epileptogenic areas; the present study will contribute to our understanding as to whether these patients are also appropriate candidates for Focal Cortex Stimulation.

Comment/Question:
In the methods section it is unclear how the EEG data will be analyzed and integrated to serve as a biomarker. Could the authors describe in a more detailed manner the "data analysis" section?

Response:
EEG recordings will be evaluated on two levels. First, the topography of epileptic discharges will be compared between time points before and after treatment with neurostimulation, focusing on the extent and propagation directions. Second, network analysis will be performed on EEG intervals without epileptic spikes to characterize changes in the epileptic networks, which may have predictive power for treatment outcomes on FCS treatment. A recent study by our group on network connectivity provides evidence that network analyses (network descriptors) have the potential to predict seizure freedom in patients undergoing epilepsy surgery [22].

The data analysis section was complemented accordingly (page 9, Data analysis), stating "Similar network analyses have already demonstrated their potential as prognostic factors for predicting seizure freedom in patients undergoing epilepsy surgery".

Comment/Question:
Could the authors also provide additional information concerning the stimulation protocol, specifically when are the patients instructed to use the additional HFS stimulation.

Response:
We do not propose or require any specific stimulation to be followed during this study, given its non-interventional design for the prospective arm. Each participating center is free to follow its routine clinical practice based on experience or to utilize the stimulation protocol outlined in the EASEE II and PIMIDES I clinical trials. A detailed description of the protocol can be found in [9, 10]. To improve clarity, we included a clear reference to this (page 7-8, Data collection), stating "Consequently, there are no prespecified follow-up visits, stimulation protocols, as treatment is solely guided by clinical need".

The stimulation protocol used in the trials:

- Stimulation 1 month post-implantation to differentiate surgery effects from stimulation outcomes.
- Combined Intermittent bursts of 100-Hz high-frequency stimulation (HFS) every 2 minutes and 20 minutes of continuous cathodal direct low stimulation (DLS) per day.
- Stimulation amplitude below the perception threshold (up to 4 mA), adjustable during follow-ups.
- Stimulation settings mostly consistent over 6 months, with minor adjustments to maintain imperceptibility.
- In the PIMIDES I trial, patients could trigger additional HFS pulses (10 to 60 seconds) when early ictal symptoms were detected.

Reviewer 2

It has been my pleasure to review this manuscript entitled Therapy response prediction of focal cortex stimulation based on clinical parameters – a multicenter, observational study protocol'. This manuscript summarizes the protocol of an ongoing study using a new type of neuromodulation device called focal cortex stimulation. This device is used in patients with focal drug resistant epilepsy and requires the device to be implanted subgaleal over the epileptogenic focus. Overall this paper is nicely written manuscript. However, additional important information should be included.

<u>Comment/Question:</u>
In the abstract, if severity of seizures are evaluated, that information should be mentioned.

<u>Response:</u>
The word "severity" has now been included as a key outcome parameter, alongside seizure frequency, in the abstract.

<u>Comment/Question:</u>
Regarding the keywords, neuromodulation should be included, as well as focal epilepsy.

<u>Response:</u>
We thank the reviewer for this suggestion. We would like to include these keywords; however, the submission portal only allows the selection of keywords from a pre-specified list, and the proposed keywords are not included there.

<u>Comment/Question:</u>
Additional information regarding the procedure, complications and side effects should be included in the introduction.

<u>Response:</u>
The implantation of the 54.4 × 54.4 mm^2 EASEE electrode subgaleally is considered to be less invasive than other long-term neuromodulatory approaches [8]. No serious adverse events have been reported during or related to the procedure, nor from the stimulation itself [9].

We have now included this information in our introduction, stating "No serious adverse events were considered to be associated with the procedure or the device at 8 months[8,9]", along with a source

on the early surgical experience with the device for readers interested in the technical details of the procedure [8] (page 5).

Comment/Question:
A clear explanation why FCS is better than open surgery or other neuromodulation devices should be mentioned, to make the case for new technologies.

Response:
The two pilot trials demonstrate a seizure reduction of ≥50% in approximately half of the trials' cohort. This is comparable to other readily available neuromodulation procedures, such as RNS and ANT-DBS. Unlike these intracranial approaches, the EASEE system offers a significantly less invasive treatment strategy for pharmacoresistant epilepsy [8]. In this context, it is more akin to the VNS device (page 5). The introduction has been updated accordingly.

We certainly do not claim that FCS is superior to open neurosurgery. Rather, we consider this to be a complementary approach for patients who are poor surgical candidates e.g. due to high risks of resections, or to patients who do not wish to undergo surgery.

Comment/Question:
Furthermore, a better justification of why the proposed stimulation paradigms are selected needs to be incorporated into the manuscript.

Response:
We do not propose or require any specific stimulation to be followed during this study, given its non-interventional design. Each participating center is free to follow its routine clinical practice based on experience or to utilize the stimulation protocol outlined in the EASEE II and PIMIDES I clinical trials. A detailed description of the protocol can be found in [9, 10]. To improve clarity, we included a clear reference to this (page 7, Data collection), stating "Consequently, there are no prespecified follow-up visits, stimulation protocols, as treatment is solely guided by clinical need".

The stimulation protocol used in the trials:

- Stimulation 1 month post-implantation to differentiate surgery effects from stimulation outcomes.
- Combined Intermittent bursts of 100-Hz high-frequency stimulation (HFS) every 2 minutes and 20 minutes of continuous cathodal direct low stimulation (DLS) per day.
- Stimulation amplitude below the perception threshold (up to 4 mA), adjustable during follow-ups.
- Stimulation settings mostly consistent over 6 months, with minor adjustments to maintain imperceptibility.
- In the PIMIDES I trial, patients could trigger additional HFS pulses (10 to 60 seconds) when early ictal symptoms were detected.

Comment/Question:
In the study population section, a clear list of exclusion criteria should be included.

Response:

These are the only criteria that apply to our study population, as approved by the ethics committees. Our non-interventional study protocol does not have any exclusion criteria by design. We would like to investigate the response to FCS in every individual implanted based on clinical decision-making. Our multicenter design seeks to capture the full spectrum of focal epilepsy and associated clinical practices.

Comment/Question:
Similarly, the duration of the follow ups for evaluating outcomes needs to be mentioned.

Response:
As the prospective arm is non-interventional, there are no prespecified follow-up visits, as to prevent any additional study related burden for the study participants. Each participating center may define its own follow-up scheme, or follow the scheme used in the EASEE II and PIMIDES I trials [9]. We updated the relevant section: "Consequently, there are no prespecified follow-up visits, stimulation protocols, as treatment is solely guided by clinical need. This approach ensures there is no additional risk or burden for the patients involved in the conduct of this study" (page 7).

Participants are followed throughout their individual stimulation treatment period up to a maximum of three years from the system activation date. This sentence was originally in the Study population section (page 7) and has now been transferred to Data collection section (page 8).

Comment/Question:
It is mentioned that patients can modify ASM during the follow up period, please justify how you will control for ASM in the evaluation of the different outcomes.

Response:
Due to the non-interventional nature of the study, no control for ASM changes is possible. However, ASM types and dosages are part of the clinical data gathered, and will be included in the multivariate and machine learning analyses. This includes categorization of ASM based on their mechanisms of action, grouping them into relevant classes such as sodium channel blockers, calcium channel blockers, GABAergic agents, and others as well as analyses of the daily defined dosages applied.

Through this approach, we aim to determine whether specific combinations of ASMs or particular groups exhibit synergistic effects on seizure reduction or other clinical outcomes.

Comment/Question:
In line 15 of study population, you mentioned this study is 'non-interventional'. A clear explanation why this study was categorized as 'non-interventional' needs to be included in the manuscript.

Response:
The retrospective arm of this study includes past data from the two completed clinical trials (EASEE II and PIMIDES I) that effectively led to CE approval of the device, and by nature is non-interventional.

In the prospective arm, participants are implanted based entirely on clinical decision-making at each site, independent of the conduct of our study. Furthermore, we rely exclusively on data collected during routine clinical practice at each participating center. There are no prespecified follow-up visits, fixed stimulation protocols, or any additional burden for the patients. Consequently, there is no

intervention associated with patient participation in this study and the collected data may be incomplete (page 7-8, Data collection).

We have adjusted the main text to improve clarity: "The retrospective arm utilizes data previously collected during the EASEE II and PIMIDES I clinical trials. In the prospective non-interventional phase, we exclusively collect data derived from routine clinical care at each site, both before and after electrode implantation. Consequently, there are no prespecified follow-up visits, stimulation protocols, as treatment is solely guided by clinical need. This approach ensures there is no additional risk or burden for the patients involved in the conduct of this study" (page 7-8, Data collection).

Comment/Question:
An interesting part of the paper is the analysis of EEG and high-density EEG. Is High-density EEG a requirement? That may influence the results.

Response:
All data of interest were specified by the clinical routine followed at the coordinating center of Freiburg (page 8, Data collection), where HD-EEG is routinely used in in both presurgical evaluations and follow-up visits.

Due to the non-interventional nature of this study, we may exclusively collect data derived from routine clinical care at each site. As a result, HD-EEG is not a requirement for any participating center. However, we will include and analyze data from any center that has incorporated HD-EEG either in their presurgical diagnostics and/or follow-up visits.

Comment/Question:
Please include how many patients will underwent to SEEG prior to FCS and how that may influence the FCS outcomes.

Response:
No invasive recordings are required for focus localization prior to implantation. Similar to the EASEE II and PIMIDES I trials, we do not exclude patients who have previously undergone sEEG monitoring. The influence of prior sEEG on FCS outcomes has not been investigated in the aforementioned clinical studies. However, our larger cohort might allow us to examine this as a separate factor.

VERSION 2 - REVIEW

**Reviewer**	**2**
**Name**	**Suller Marti, Ana**
**Affiliation**	**Western University, Clinical Neurological Sciences**
**Date**	**07-Nov-2024**
**COI**	

Thank you for the effort devoted to improving the manuscript. A few issues have been addressed and others still remain unclear.

1. You added in the introduction that ‘no serious adverse events were considered ‘. Please also include mild and moderate side effects and complications. Ideally, you should compare the % of side effects and complications to other neuromodulation devices.

2. In the same paragraph, you stated that ‘The efficacy is comparable’. Please include the values of responder rates of the other neuromodulation devices.

3. The first 2 bullet points are not clear and they should be rephrased.

4. The last bullet point includes ‘selected cases’. Please specify the ‘selected cases’.

5. Inclusion criteria have been included. However, exclusion criteria has not been included. Please include an exclusion criteria statement.

6. The duration of follow up, frequency of visits, should be mentioned.

7. One of the statements is to minimize the ‘burden of the patients’. However, you are requesting a seizure diary. Please confirm how often the patients need to complete the diary.

8. The analysis of the SEEG data remains unclear. The use of two different methods for MR negative or MR lesional are proposed. How many lesional cases are you expecting to obtain? Further details in regards the methods should be provided and reference to the methodology used is required.

9. In the response to the comments, the stimulation protocol was included. However, this information was not included in the text. Please include it in the manuscript.

10. How are you going to control for changes in antiseizure medications?

11. The limitations of the study should be included.

VERSION 2 - AUTHOR RESPONSE

Reviewer 2

1. <u>Comment/Question:</u>

You added in the introduction that ‘no serious adverse events were considered ‘. Please also include mild and moderate side effects and complications. Ideally, you should compare the % of side effects and complications to other neuromodulation devices.

<u>Response:</u>
So far, published data comprise 38 patients, who have been implanted with the EASEE device, 5 of whom outside of the initial clinical trials [8,9]. Both works report that any serious adverse events were considered unrelated to the surgical implantation, device, or stimulation.

We have updated the relevant section by including the mild and moderate adverse events:

"No serious adverse events (SAE) were considered to be associated with the surgical procedure, the device, or stimulation at 8 months [9]. Similarly, no surgical SAEs were reported for five patients implanted outside these trials [8]. Mild adverse events considered related to implantation included transient pain or tingling at the skin incision sites. Other reported adverse events included five patients who experienced an increase in seizure frequency, one case of status epilepticus in a patient with a prior history before implantation, the onset of depressive symptoms in a patient with a preexisting condition, and one case attributed to COVID-19 restrictions [9]".

2. <u>Comment/Question:</u>
In the same paragraph, you stated that 'The efficacy is comparable'. Please include the values of responder rates of the other neuromodulation devices.

<u>Response:</u>
At one-year follow-up, the VNS responder rate ranges from 31% to 49%, with a median or mean seizure reduction of 26% to 52% [7]. For ANT-DBS, the SANTE study reported a responder rate of 43% with a median seizure reduction of 41% at 12 months, while the European MORE registry reported a responder rate of 32.3% and a median seizure reduction of 33.1% at 24 months [11,12]. For RNS, the responder rate after 12 months was also 43%, with a median seizure reduction of 44% [13,14]. Relevant adverse events include hoarseness (29%) and paresthesia (12%) for VNS, depression (15%), memory impairment (13%), and intracranial hemorrhage (5%) for ANT-DBS. For RNS, intracranial hemorrhage was reported in 12% of patients, and headaches in 11% [15].

This information has now been included in the introduction.

3. <u>Comment/Question:</u>
The first 2 bullet points are not clear and they should be rephrased.

<u>Response:</u>
We would like to clarify:

1. "Multicenter, non-interventional design to cover the spectrum of focal epilepsy and clinical practices." This bullet point refers to the study design, which enables us to observe all clinical practices associated with FCS treatment across multiple centers—from the initial implantation indication to the chosen stimulation parameters.
   We have revised to: "Multicenter, non-interventional study design assessing clinical practice and outcomes of Focal Cortex Stimulation."

2. "Implementation of explainable and interpretable state-of-the-art machine learning-based analytics for therapy response prediction". We understand your concerns

regarding the clarity of these terms, as they lack universally accepted definitions and mathematical rigor.[1] Nonetheless, they are widely used in machine learning to describe how and why a model arrives at a specific output in ways that are understandable to humans. Both qualities are crucial for any medical application of machine learning.

We have revised the bullet point to: "Implementation of interpretable state-of-the-art machine learning techniques for therapy response prediction".

4. Comment/Question:

The last bullet point includes 'selected cases'. Please specify the 'selected cases'.

Response:

We have eliminated this bullet point and replaced it with "Limited validity of self-reported seizure diaries", to better reflect the limitations associated with their use.

5. Comment/Question:

Inclusion criteria have been included. However, exclusion criteria has not been included. Please include an exclusion criteria statement.

Response:

These are the only criteria that apply to our study population, as approved by multiple ethics committees. There are no exclusion criteria, as this non-interventional study is designed to reflect the clinical practice of FCS use in the participating centers.

6. Comment/Question:

The duration of follow up, frequency of visits, should be mentioned.

Response:

We kindly refer to the "Data collection" section, page 7 and to our previous response:

"As the prospective arm is non-interventional, there are no prespecified follow-up visits, as to prevent any additional study related burden for the study participants. Each participating center may define its own follow-up scheme, or follow the scheme used in the EASEE II and PIMIDES I trials [9]. We updated the relevant section: "Consequently, there are no prespecified follow-up visits, stimulation protocols, as treatment is solely guided by clinical need. This approach ensures there is no additional risk or burden for the patients involved in the conduct of this study" (page 7).

Participants are followed throughout their individual stimulation treatment period up to a maximum of three years from the system activation date. This sentence was originally in the

Study population section (page 7) and has now been transferred to Data collection section (page 8)."

7. <u>Comment/Question:</u>
One of the statements is to minimize the 'burden of the patients'. However, you are requesting a seizure diary. Please confirm how often the patients need to complete the diary.

<u>Response:</u>
Seizure diaries are the clinical standard for assessing the severity and course of epilepsy worldwide, including in the clinical practices of all participating centers. As this is a non-interventional study, we do not require any actions from participants beyond routine clinical care at each site, nor do we make any specific recommendations.
Any recommendations regarding how patients complete their diaries are made by the participating clinicians and are independent of the conduct of this study.

8. <u>Comment/Question:</u>
The analysis of the SEEG data remains unclear. The use of two different methods for MR negative or MR lesional are proposed. How many lesional cases are you expecting to obtain? Further details in regards the methods should be provided and reference to the methodology used is required.

<u>Response:</u>
We would like to clarify that sEEG data is not included in the analyses for this study. The EEG analysis is limited to scalp EEG for both lesional and non-lesional patients.
We kindly refer to our previous response:
"No invasive recordings are required for focus localization prior to implantation. Similar to the EASEE II and PIMIDES I trials, we do not exclude patients who have previously undergone sEEG monitoring. The influence of prior sEEG implantation on FCS outcomes has not been investigated in the aforementioned clinical studies. However, our larger cohort might allow us to examine this as a separate factor".

In the two completed clinical trials, 10 out of 33 patients had non-lesional epilepsy. Since device implantation in this study is conducted according to the clinical judgment of the participating centers, the percentage of non-lesional patients is not predefined and can only be analyzed retrospectively.

The proposed methods rely on a novel MR sequence developed by the Neuroradiology Department at Freiburg University Medical Center, which is currently undergoing validation. This MR sequence enables the spatial definition of the EASEE electrode relative to the lesion

or region of interest identified through EEG in non-lesional cases. The magnitude of the stimulation field is informed by experimental studies using the EASEE electrode in phantom models. These enable the simulation of the electrical field, which can be applied to each individual's MRI to model the field strength in the regions of interest. A detailed description of this process is beyond the scope of the current protocol and may be presented in a separate publication.

Furthermore, we have included a reference for the methodology used for the assessment of the topography of epileptiform activity [26].

9. Comment/Question:

In the response to the comments, the stimulation protocol was included. However, this information was not included in the text. Please include it in the manuscript.

Response:

Since this protocol was used exclusively in the two completed clinical trials (EASEE II and PIMIDES I) and does not apply to the prospective arm, including it in the main text may lead to misconceptions rather than enhance clarity. We neither require nor recommend that any center use it; each participating center is free to follow its routine clinical practice.
Nevertheless, we have made several references to the publication, where it is publicly available.

10. Comment/Question:

How are you going to control for changes in antiseizure medications?

Response:

Given the non-interventional character of the study, there are no recommendations as to the conduct of antiseizure medication. Changes in antiseizure medication will, however be considered as a covariate in the outcome assessment.

We kindly refer to our previous response:

"Due to the non-interventional nature of the study, no control for ASM changes is possible. However, ASM types and dosages are part of the clinical data gathered, and will be included in the multivariate and machine learning analyses. This includes categorization of ASM based on their mechanisms of action, grouping them into relevant classes such as sodium channel blockers, calcium channel blockers, GABAergic agents, and others as well as analyses of the daily defined dosages applied.

Through this approach, we aim to determine whether specific combinations of ASMs or particular groups exhibit synergistic effects on seizure reduction or other clinical outcomes".

11. Comment/Question:
The limitations of the study should be included.

Response:
There are two clear limitations in this study: First, outcomes are documented according to the clinical standard of self-reported events in a seizure diary, which are known to be of limited validity, although they are considered the gold-standard procedure for any ASM or stimulation device trial. Second, the non-interventional nature of this study prevents direct control over many potential factors affecting outcomes, including changes in ASM. Additionally, follow-up visits are not prespecified, and data may be incomplete if specific information is not part of the routine clinical practice at any participating center. However, the inclusion of multiple participating centers may allow to capture the broad spectrum of clinical practices.
These points are included either in the "STRENGTHS AND LIMITATIONS OF THIS STUDY" section or in the "Data Collection" section.

REFERENCE:

1. Marcinkevičs R, Vogt JE. Interpretable and explainable machine learning: A methods-centric overview with concrete examples. WIREs Data Min Knowl Discov. 2023;13(3):e1493.

VERSION 3 - REVIEW

**Reviewer**	**2**
**Name**	**Suller Marti, Ana**
**Affiliation**	**Western University, Clinical Neurological Sciences**
**Date**	**22-Dec-2024**
**COI**	

No further concerns are needed to be addressed.
